# Echo-Doppler–derived indexes of ventricular stiffness and ventriculo-arterial interaction as predictors of new-onset atrial fibrillation in patients with heart failure

**DOI:** 10.1186/s12947-016-0050-y

**Published:** 2016-02-04

**Authors:** Ji Hyun Yoon, Myung-Hyun Kim, Hyemoon Chung, Eui-Young Choi, Pil-Ki Min, Young Won Yoon, Byoung Kwon Lee, Bum-Kee Hong, Se-Joong Rim, Hyuck Moon Kwon, Jong-Youn Kim

**Affiliations:** Heart Center, Gangnam Severance Hospital, Department of Internal Medicine, Yonsei University College of Medicine, Gangnam Severance Hospital, 211 Eonju-ro, Gangnam-gu, Seoul 135-720 South Korea

**Keywords:** Left ventricular stiffness, Atrial fibrillation, Heart failure, Diastolic dysfunction

## Abstract

**Background:**

Atrial fibrillation (AF) is common in patients with heart failure (HF) and worsens their prognosis. Vulnerability to changes in loading is an important factor in the development of AF and is strongly influenced by ventricular stiffness and ventriculo-arterial interaction. The aim of this study was to investigate predictors of AF development in patients with HF.

**Methods:**

We studied 349 patients with stable HF. The following parameters of ventricular stiffness and ventriculo-arterial interaction were derived from echo-Doppler measurements: left ventricular (LV) diastolic elastance (Ed), effective arterial elastance (Ea), LV end-systolic elastance (Ees) and ventricular–vascular coupling index (VVI).

**Results:**

AF occurred in 57 (16.3 %) patients over a median follow up of 30.3 months. Echo-Doppler–derived parameters of ventricular stiffness and ventriculo-arterial interaction were closely associated with HF severity. Ed was independently associated with AF after adjustment for age, hypertension, diabetes mellitus, and left atrial volume index (hazard ratio [HR] 5.49, *p* = 0.018). Ea and VVI were also associated with new-onset AF (HR 1.66, *p* = 0.027, and HR 1.06, *p* = 0.001, respectively).

**Conclusions:**

Echo-Doppler indexes of ventricular stiffness are closely associated with HF severity. LV diastolic elastance (Ed) is the strongest predictor of new-onset AF in HF patients.

**Electronic supplementary material:**

The online version of this article (doi:10.1186/s12947-016-0050-y) contains supplementary material, which is available to authorized users.

## Background

Heart failure (HF) and atrial fibrillation (AF) each affect 1–2 % of the general population and the prevalence of both increases with age [1, 2]. AF is the most common sustained arrhythmia and occurs in 15 to 30 % of patients with HF [3, 4]. Since AF is a marker of increased cardiovascular mortality and morbidity in patients with HF [5, 6], it is important to investigate the predictors of AF occurrence in these patients. The development of AF in HF appears to be a multifactorial process, involving structural and neurohormonal processes and electrical remodeling [7]. The heart’s vulnerability to changes in loading affects the prognosis of HF and the development of AF. As this vulnerability is affected by ventricular stiffness and ventriculo-arterial interaction [8, 9], it is reasonable to hypothesize that those factors are closely associated with the development of AF. However, the relationship has still not been determined in HF patients. Therefore, the aim of this study was to investigate indexes of ventricular stiffness and ventriculo-arterial interaction as predictors for the development of AF in patients with HF.

## Methods

### Study populations

We retrospectively investigated 349 consecutive patients (238 men; age 62 ± 15 years) with stable HF with normal sinus rhythm who had undergone echocardiography between January 2007 and December 2008 in our institution. Reduced left ventricular (LV) contractility was defined as either a reduced ejection fraction (EF) (EF < 40 %) or a borderline EF (40 % ≤ EF < 50 %), based on two-dimensional echocardiography [10]. Exclusion criteria were (1) muscular dystrophy, (2) more than moderate valvular disease, (3) previously documented AF or atrial flutter on the electrocardiogram (ECG) or Holter monitoring, (4) significant lung disease and (5) a New York Heart Association functional classification III or IV.

We reviewed medical history, physical examinations, laboratory tests, medication records, chest radiographs, 12-lead ECG, and echocardiography findings in all patients. We followed the study population for the development of AF via a review of their medical records. New-onset AF was defined as the first episode of paroxysmal or persistent AF, as documented by resting ECG or 24-h Holter monitoring, during the follow-up period. When the patient presented symptoms such as palpitations or dizziness, 24-h Holter monitoring was performed. Paroxysmal AF was defined as AF that terminated spontaneously within seven days, and persistent AF as continuous AF that was sustained beyond seven days [5]. Study protocols were approved by our institutional review board, and informed consent from patients was waived due to retrospective nature of the study.

### Conventional echocardiography

Two-dimensional, pulsed-Doppler, and tissue-Doppler echocardiographic examinations were performed using an appropriate ultrasound device (Sonos 7500, Philips, or Vivid 7, GE healthcare). Standard 2-dimensional measurements—LV diastolic and systolic dimension, ventricular septum and posterior wall thicknesses, left atrial (LA) volume, and LV outflow tract diameter—were obtained, based on the recommendations of the American Society of Echocardiography [6]. LVEF was calculated from minimal (LV end-systole just before mitral valve opening) and maximal (LV end-diastole at the start of the QRS) LV volumes, which were obtained using a modified Quinones method [5]. LA volume was determined by the prolate ellipsoid formula and was indexed to the body surface area [6]. Peak velocities of early diastolic filling (E) and late diastolic filling (A) were measured by the pulsed-wave Doppler method at the level of the mitral valve leaflet tips. Peak early diastolic velocity (e’) and late diastolic velocity (a’) were measured by tissue Doppler imaging from the septal mitral annulus. The E/A and E/e’ ratios were calculated to estimate the LV filling pressure [7]. Echocardiographic data were analyzed by two cardiologists who were blinded to the patients’ other information.

### Echo-Doppler–derived parameters of ventricular stiffness and ventriculo-arterial interaction

For the evaluation of the vulnerability of the LV, we measured ventricular stiffness and end-systolic ventriculo-arterial interaction using echo-Doppler [8, 9]. Ventricular diastolic elastance (Ed) was calculated as E/e’ divided by stroke volume [11, 12]. Ventricular end-systolic elastance (Ees) was required to describe the contractility of the LV and was calculated based on pulsed-wave Doppler echocardiography of trans-LV outflow tract (LVOT) as peak velocity divided by acceleration time [13]. Peak velocity of LVOT was measured at the point of maximal velocity and acceleration time was measured as the time from the onset to the peak velocity. Effective arterial elastance (Ea) provides an integrative measure of arterial load and was defined as the ratio of end-systolic pressure to stroke volume where end-systolic pressure was calculated as (2 × systolic blood pressure + diastolic blood pressure)/3 [11, 12]. To evaluate the interaction of the ventricular and arterial systems, the ventricular–vascular coupling index (VVI) was calculated as the ratio Ea/Ees [14, 15].

### Statistical analysis

Baseline characteristics were summarized for all participants using standard descriptive statistics. Continuous variables are presented as mean ± standard deviation and categorical variables as number and percentage. Baseline characteristics of groups were compared using independent t-tests for continuous variables and chi-square tests for categorical variables. Variables without normal distribution were presented as median (inter-quartile range) and Mann-Whitney *U* test were used for their comparisons.

Linear regression analyses were performed to determine the association between echo-derived parameters and the HF severity, estimated by EF and levels of log-transformed brain natriuretic peptide (BNP). To achieve a constant variance, BNP values were transformed logarithmically. Cox proportional hazard models were used to determine the association between Ed, Ees, Ea, and VVI, and the development of new-onset AF. Adjustment variables were selected based on the differences between two groups (AF vs. sinus rhythm) and clinical rationale; they included age, LA volume index(LAVI), and underlying disease. We also used receiver operating characteristic curves to derive cutoff values for predictors. Time-to-event data are reported and displayed using the Gehan’s generalized Wilcoxon method, with comparisons between groups performed using the log-rank testDifferences were considered statistically significant if the *p*-value was <0.05 when using two-sided tests. All statistical analyses were performed using SPSS 18.0 (SPSS Inc., Chicago, Illinois).

## Results

Patient characteristics and echocardiographic parameters are presented in Table 1. New-onset AF developed in 57 of the 349 patients studied (16.3 %). The mean age of the study population was 62 ± 15 years and 238 (68 %) were male. At the beginning of the study, all patients were in normal sinus rhythm. The median follow-up period was 30.3 months. Anticoagulation medication was administered to all patients who developed AF and had appropriate indications.

**Table 1:** Intergroup comparison of clinical characteristics and echocardiographic parameters

Characteristic	Sinus rhythm	New-onset AF	*p*-value
	(*n* = 292)	(*n* = 57)	
Age (years)	61.2 ± 15.7	67.7 ± 12.8	*0.003
Male, n (%)	203 (69.5)	35 (61.4)	0.229
Body surface area (m^2^)	1.71 ± 0.23	1.68 ± 0.19	0.320
SBP (mmHg)	122.4 ± 23.3	115.4 ± 25.2	0.094
DBP (mmHg)	74.1 ± 16.0	68.1 ± 14.7	*0.032
Hypertension, n (%)	94 (32.2)	28 (49.1)	*0.014
Diabetes mellitus, n (%)	52 (17.8)	19 (33.3)	*0.008
Current smoker, n (%)	57 (19.5)	12 (21.1)	0.791
Ischemic CMP, n (%)	164 (56.2)	32 (56.1)	0.997
Chronic kidney disease, n (%)	29 (12.0)	10 (18.5)	0.264
BUN (mg/dL)	21.7 ± 14.9	24.4 ± 21.5	0.259
Creatinine (mg/dL)	1.4 ± 1.5	2.0 ± 2.3	0.095
BNP (pg/ml)	Median: 216.0 (81.8–749)	Median: 566.0(180.5-1302.5)	*0.011
Medications			
Antiplatelet agent, n (%)	218 (74.7)	46 (80.7)	0.400
RAS blockade, n (%)	243 (83.2)	49 (86.0)	0.608
Diuretics, n (%)	131 (44.9)	42 (73.7)	* < 0.001
Beta blockade, n (%)	191 (65.4)	31 (54.4)	0.114
Calcium channel blockade, n (%)	88 (30.1)	21 (36.8)	0.318
Statin, n (%)	152 (52.1)	26 (45.6)	0.374
Echocardiographic parameters			
Ejection fraction (%)	36.9 ± 9.4	35.3 ± 10.3	0.249
LVEDD (mm)	52.4 ± 7.1	52.4 ± 7.9	0.964
LVESD (mm)	42.0 ± 8.1	42.3 ± 8.7	0.814
LA-AP (mm)	37.8 ± 7.4	40.3 ± 7.5	*0.023
LA-ML (mm)	43.9 ± 7.5	47.8 ± 8.7	*0.001
LA-SI (mm)	52.8 ± 8.7	57.5 ± 9.7	* < 0.001
LA volume index (mm^3^/m^2^)	28.4 ± 12.7	36.1 ± 14.8	* < 0.001
LV mass index (g/m^2^)	73.8 ± 27.6	74.8 ± 18.9	0.738
E velocity (m/s)	0.65 ± 0.23	0.79 ± 0.30	* < 0.001
A velocity (m/s)	0.72 ± 0.22	0.71 ± 0.25	0.706
E/A	1.02 ± 0.63	1.22 ± 0.75	*0.036
E/DT (cm/s)	0.39 ± 0.27	0.55 ± 0.37	*0.003
e’ (m/s)	0.05 ± 0.02	0.04 ± 0.02	0.278
a’ (m/s)	0.08 ± 0.02	0.07 ± 0.03	*0.001
S’ (m/s)	0.05 ± 0.02	0.05 ± 0.02	0.223
E/e’	15.2 ± 6.8	19.1 ± 7.2	* < 0.001
Ed (1/ml)	0.36 ± 0.22	0.47 ± 0.27	*0.009
Ees (m/s)	1.02 ± 0.24	1.11 ± 0.37	0.195
Ea (mmHg/ml)	2.67 ± 0.96	2.75 ± 1.43	0.717
VVI	25.5 ± 9.1	32.1 ± 16.8	0.115

*Abbreviations*: *AF* atrial fibrillation, *CMP* cardiomyopathy, *BUN* blood urea nitrogen, *RAS* renin-angiotensin system, *LVEDD* left ventricular end-diastolic dimension; *LVESD*, left ventricular end-systolic dimension; *LA* left atrial; *AP* anteroposterior; *ML* mediolateral; *SI* Superoinferior; *DT* Deceleration time; *Ed* left ventricular (LV) end-diastolic elastance; *Ees*, LV end-systolic elastance; *Ea*, effective arterial elastance; *VVI*, ventricular-vascular coupling index

* *p* < 0.05

The new-onset AF group was older (67.7 ± 12.8 vs. 61.2 ± 15.7, *p* = 0.003) and had a higher prevalence of hypertension (49.1 % vs. 32.2 %, *p* = 0.014) and diabetes (33.3 % vs. 17.8 %, *p* = 0.008). The rate of ischemic cardiomyopathy did not differ between the two groups. The new-onset AF group used more diuretics (73.7 % vs. 44.9 %, *p* < 0.001) than the sinus rhythm group (Table 1). This finding suggests that patients with AF may have more symptoms of heart failure and more volume overloading conditions. The median BNP level was higher (216.0 vs. 566.0, *p* = 0.011) in patients with AF.

There was no difference in LV end-diastolic diameter (52.4 ± 7.1 vs. 52.4 ± 7.9 mm, *p* = 0.964), LV systolic function (EF: 36.9 ± 9.4 % vs. 35.3 ± 10.3 %, *p* = 0.249), LV mass index (73.8 ± 27.6 vs. 74.8 ± 18.9 g/m^2^, *p* = 0.738), A (0.72 ± 0.22 vs. 0.71 ± 0.25 m/s, *p* = 0.706), or e’ (0.71 ± 0.25 vs. 0.04 ± 0.02 m/s, *p* = 0.278). However, the new-onset AF group had a larger LA volume index (36.1 ± 14.8 vs. 28.4 ± 12.7 mL/m^2^, *p* < 0.001), a higher E (0.79 ± 0.30 vs. 0.65 ± 0.23 m/s, *p* < 0.001), and a higher E/e’ ratio (19.1 ± 7.2 vs. 15.2 ± 6.8, *p* < 0.001). A’ was significantly lower in the new-onset AF group (0.07 ± 0.03 vs. 0.08 ± 0.02 m/s, *p <* 0.001) (Table 1). Among the echo-derived Doppler indexes, Ed was significantly higher (0.47 ± 0.27 vs. 0.36 ± 0.22 /mL, *p* = 0.009) in the new-onset AF group. Ees, Ea, and VVI did not differ significantly between the two groups.

In linear regression, EF measured by echocardiography was significantly correlated with Ed (β: -0.496, *p* < 0.001) and Ea (β: -0.314, *p* < 0.001). In addition, BNP level was significantly correlated with Ed (β: 0.609, *p* < 0.001), Ees (β: 0.218, *p* =0.020) and Ea (β: 0.212, *p* =0.031) (Table 2). These findings suggest that there are significant relationships between HF severity, estimated by EF and BNP level, and echo-derived Doppler parameters that reflect ventricular stiffness (Ed) and arterial load (Ea).

**Table 2:** Association of HF severity with ventricular stiffness and ventriculoarterial interaction index by univariate linear regression

Ejection fraction			BNP(Log-transformed)	
	ß	*p*-value	ß	*p*-value
Ed (1/ml)	−0.496	* < 0.001	0.609	^*^ <0.001
Ees (m/s)	0.001	0.991	0.218	^*^ 0.020
Ea (mmHg/ml)	−0.314	* < 0.001	0.212	^*^ 0.031
VVI	−0.190	0.064	0.040	0.735

*Ed* left ventricular (LV) diastolic elastance; *Ees* LV end-systolic elastance; *Ea* effective arterial elastance; *VVI* ventricular-vascular coupling index

Over a median follow up of 30.3 months, new-onset AF developed in 57 patients (16.3 %). Lower EF was significantly associated with new-onset AF (hazard ratio [HR] 0.97, *p* = 0.025). After adjustment for age, hypertension, diabetes mellitus, use of diuretics and LAVI, EF was not correlated with AF (HR 0.98, *p* = 0.250). Ed was also significantly associated with new-onset AF, with a greater than 10-fold risk in an unadjusted model (HR 10.40, *p* < 0.001). This association remained significant after adjustment for age, hypertension, diabetes mellitus, use of diuretics and LAVI, (HR 5.49, *p* = 0.018). Ea and VVI were associated with new-onset AF after adjustment for age, hypertension, diabetes mellitus, use of diuretics and LAVI (HR 1.66 and 1.06, *p* = 0.026 and 0.001, respectively) (Table 3). For subgroup analysis, we devided our study population into two groups: (1) heart failure with reduced EF (HFrEF, EF < 40 %) and (2) heart failure with preserved EF (HFpEF, EF ≥ 40 %). In patients with HFrEF, Ea and VVI were associated with new-onset AF after adjustment for age, hypertension, diabetes mellitus, use of diuretics and LAVI (HR 1.73 and 1.04, *p* = 0.045 and 0.035, respectively) (Additional file 1). Whereas, there was no independent parameter of LV stiffness that associated with new-onset AF in patients with HFpEF.

**Table 3:** Association between echocardiographic parameters and new-onset atrial fibrillation

Unadjusted			Adjusted	
	Hazard ratio	*p*-value	Hazard ratio	*p*-value
Ejection fraction (%)	0.97	*0.025	0.98	0.250
Ed (1/ml)	10.40	* < 0.001	5.49	*0.018
Ea (mmHg/ml)	1.70	0.011	1.66	*0.027
Ees (m/s)	1.58	0.497	0.95	0.940
VVI	1.04	0.004	1.06	*0.001

Adjusted for age, hypertension, DM, use of diuretics and left atrial volume index

*Ed* left ventricular (LV) diastolic elastance; *Ea* effective arterial elastance; *Ees* LV end-systolic elastance; *VVI* ventricular-vascular coupling index

*p < 0.05

**Fig. 1:**
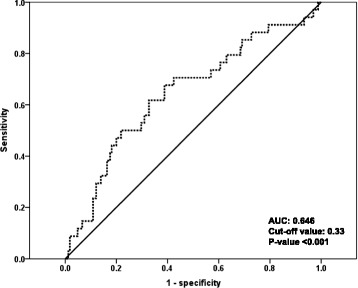
Receiver operating characteristic (ROC) curve for ventricular diastolic elastance (Ed)

**Fig. 2:**
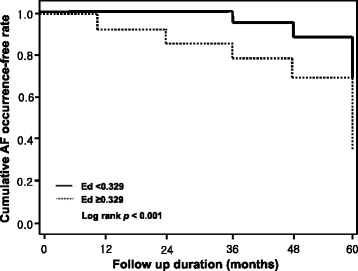
Cumulative AF occurrence free rate in relation to Ed (*p* < 0.001)

Further evaluation was performed to determine the power of Ed in predicting AF occurrence. The optimal cutoff value to predict AF was an Ed value of 0.33, with corresponding sensitivity and specificity values of 71 and 58 %, respectively (receiver operating characteristic curve: area under curve [AUC] = 0.646, *p* < 0.001) (Fig. 1). Figure 2 shows the cumulative survival curves for AF occurrence. The cumulative survival rate free of AF occurrence was significantly higher in patients with higher Ed (≥0.33) than in patients with lower Ed (<0.33) (*p* < 0.001).

## Discussion

The principle findings of this study are as follows: (1) Ventricular stiffness and ventriculo-arterial interaction could be measured by echo Doppler, and (2) Ed, Ea and VVI were closely correlated with new-onset AF in patients with HF.

### The variable effect of AF on patients with HF

AF is the most common arrhythmia [4, 9] and further aggravates symptoms in patients with HF [9, 16]. AF occurs in 10–20 % of mild to moderate cases of HF and in up to 50 % of patients with more advanced HF. The prevalence of AF generally depends on the severity of the underlying HF [17, 18]. In this study, only ambulatory patients with stable HF were enrolled and new-onset AF developed in 16.3 % of patients.

The new-onset AF group was receiving diuretics at a statistically significantly higher rate (73.7 % vs. 44.9 %, *p* < 0.001). This finding suggests that these patients were clinically identified as having volume overload or elevated LV filling pressure and were being treated as such. Recently, Cambell et al. showed that renal impairment was strong predictor of AF in patients with HF and should be regularly screened. In our study, prevalence of chronic kidney disease was not different between AF group and sinus rhythm group.

While LV systolic function was similar between the two groups, patients with new-onset AF had significantly higher LAVI, E/e’, and Ed. These findings suggest that atrial dilation and reduced LV relaxation are related to the development of AF and could be explained by diastolic dysfunction.

### The correlation between AF and diastolic dysfunction

Diastolic dysfunction, like systolic dysfunction, is known to be associated with the development of AF [11, 13]. The presence of diastolic dysfunction is also a strong predictor of AF occurrence and is closely associated with a poor prognosis in patients with HF [19]. Diastolic dysfunction leads to elevated LV filling pressures and atrial remodeling. The relationship between atrial remodeling and AF has been investigated in many studies. Diastolic dysfunction with an increase in LV filling pressures causes atrial remodeling and is associated with a more than 5-fold increased risk of AF occurrence compared with normal diastolic function [20].

### The assessment of diastolic dysfunction by echocardiography

LV diastolic filling pressure can be measured by Doppler echocardiography. Mitral inflow velocity (E) correlates well with LV filling pressure in heart failure; however, myocardial relaxation and filling pressure could affect the mitral E velocity. The mitral e’ velocity reflects relaxation of the myocardium and the E/e’ ratio correlates well with LV filling pressure or pulmonary capillary wedge pressure [21–23]. In this study, the AF group had significantly higher E/e’ values compared with patients in normal sinus rhythm.

In HF patients, increased ventricular stiffness induces diastolic dysfunction, thereby causing an elevation of LV filling pressure and increasing vulnerability to an acute loading change [14]. E/e’ is significantly correlated with LV filling pressure [22], while stroke volume can be used as an indicator of LV filling volume. Thus, combining these parameters, dividing E/e’ by stroke volume, represents the ratio of LV filling pressure to filling volume and can be used as the Ed [11]. We used Ed for the evaluation of ventricular stiffness in this study. We found that Ed was significantly correlated with EF and BNP, which represent HF severity. Furthermore, a higher Ed was independently associated with AF, after adjustment for age, hypertension, diabetes mellitus, use of diuretics and LAVI. In addition, using receiver operating characteristic analysis, we were able to determine a cutoff value for LV filling pressure/volume, as represented by the Ed value. According to our results, the optimal cutoff value of Ed for the prediction of new-onset AF was 0.33, with a sensitivity of 71 % and a specificity of 58 %.

### Ventriculo-arterial interaction

Increased afterload is also important in the pathophysiology of HF [24]. The Ea was used as the total arterial afterload and can be measured noninvasively [11, 12]. In addition, the Ees is related to LV contractility, independently of loading conditions [25]. The combination of these parameters, called VVI, is the ratio Ea/Ees and is used as an index of ventriculo-arterial coupling, as previously described [14]. In our study, we found that Ea was closely related to HF severity. Interestingly, our study showed that Ea and VVI, as well as Ed, were predictors of new-onset AF in patients with HF, whereas Ees was not. These results suggest that ventricular stiffness and ventriculo-arterial coupling are important predictors of AF occurrence in HF patients. In addition, ventricular stiffness and ventriculo-arterial coupling are more relevant than LV contractility alone.

### Limitations

The present study had some limitations. First, since it was a single-center study with a retrospective design, only limited data analysis was possible and follow-up durations were arbitrary. Second, the occurrence of new-onset AF might have been underestimated. Capturing AF is notoriously difficult, unless careful transtelephonic monitoring or regular Holter recordings can be performed. We know that relying on symptomatic presentation will underestimate the occurrence of AF, given that much of AF is asymptomatic. There is no way of knowing whether patients in the “sinus rhythm” group actually had paroxysmal AF or not. Finally, the male predominance in the study patients was a potential limitation, in that it did not reflect the general clinical HF population; however, this is a common failing shared with many other contemporary HF studies.

## Conclusions

Echo-Doppler–derived indexes of ventricular stiffness and ventriculo-arterial coupling are associated with HF severity and are important predictors of new-onset AF in patients with HF. Therefore, HF patients with increased ventricular stiffness may need close observation for the development of AF, using techniques such as frequent Holter monitoring.

## Additional file

Additional file 1:
**Association between echocardiographic parameters and** **new-onset** **atrial fibrillation in patients with heart failure with reduced ejection fraction.** (DOCX 16 kb)
