# Supplementary material for: Echo-Doppler–derived indexes of ventricular stiffness and ventriculo-arterial interaction as predictors of new-onset atrial fibrillation in patients with heart failure
Source: Cardiovasc Ultrasound. 2016 Feb 4;14:7. doi: 10.1186/s12947-016-0050-y (PMC4743395; doi:10.1186/s12947-016-0050-y)
Supplement: Additional file 1: — Association between echocardiographic parameters and new-onset atrial fibrillation in patients with heart failure with reduced ejection fraction. (DOCX 16 kb) [file 12947_2016_50_MOESM1_ESM.docx]

**Additional file 1.** Association between echocardiographic parameters and new-onset atrial fibrillation in patients with heart failure with reduced ejection fraction.

| **Unadjusted** | | | | **Adjusted** | |
| --- | --- | --- | --- | --- | --- |
|  | Hazard ratio | | p-value | Hazard ratio | p-value |
| Ejection fraction (%) | | 0.94 | *0.009 | 0.95 | 0.056 |
| Ed (1/ml) | | 7.28 | *0.008 | 3.67 | 0.142 |
| Ea (mmHg/ml) | | 1.70 | *0.018 | 1.73 | *0.045 |
| Ees | | 3.84 | *0.050 | 3.10 | 0.136 |
| VVI | | 1.04 | *0.030 | 1.04 | *0.035 |

Adjusted for age, hypertension, DM, diuretic therapy and left atrial volume index.

Ed, left ventricular (LV) diastolic elastance; Ea, effective arterial elastance; Ees, LV end-systolic elastance; VVI, ventricular-vascular coupling index
